# Supplementary material for: Single-molecule analysis of specificity and multivalency in binding of short linear substrate motifs to the APC/C
Source: Nat Commun. 2022 Jan 17;13:341. doi: 10.1038/s41467-022-28031-2 (PMC8764033; doi:10.1038/s41467-022-28031-2)
Supplement: Supplementary file 1 — Supplementary Information [file 41467_2022_28031_MOESM1_ESM.pdf]

Supplementary Information

Single-molecule analysis of specificity and multivalency in binding of short linear substrate motifs to the APC/C

Nairi Hartooni, Jongmin Sung, Ankur Jain, and David O. Morgan

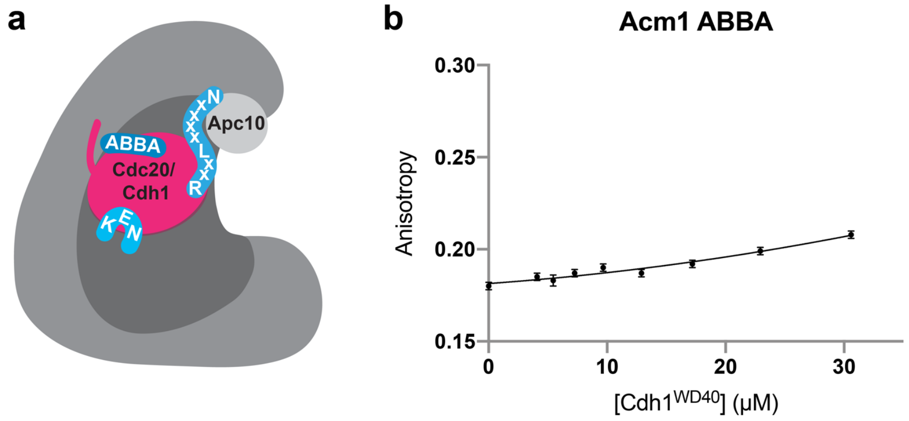

Supplementary Fig. 1: Anisotropy with Acm1 ABBA degron peptide
a, Cartoon summarizing the interactions of activated APC/C with the three major degrons found in APC/C substrates. **b**, Fluorescence anisotropy analysis of Cy5-labeled Acm1 ABBA degron peptide (SKAAQFMLYEETAEERNI-K[Cy5]; 10 nM) binding to $Cdh1^{WD40}$. Data points represent mean ± SE (n = 10 reads per reaction). Source data are provided as a Source Data file.

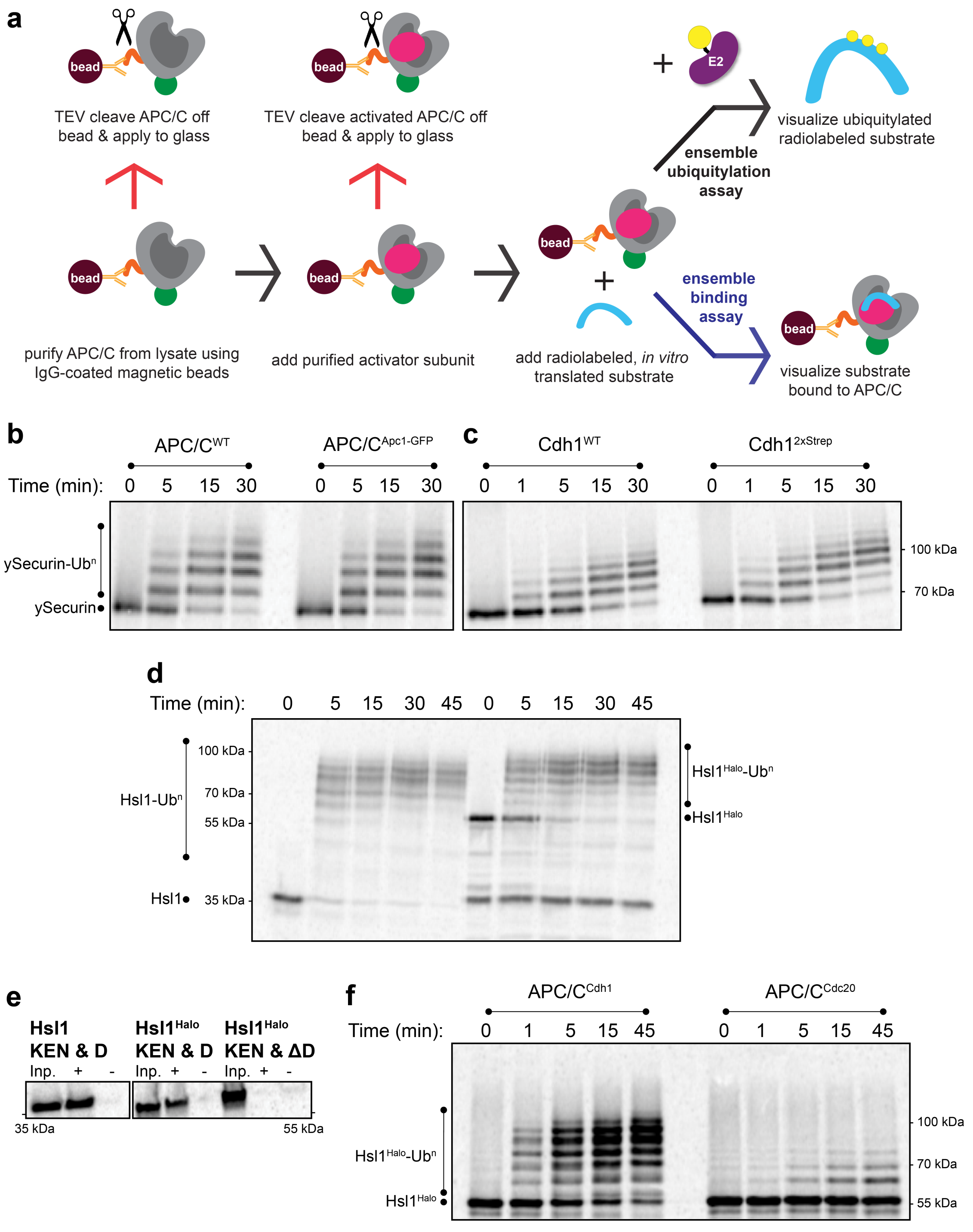

Supplementary Fig. 2: APC/C activity with tagged substrates
a, Methods for analysis of APC/C activity and substrate binding. **b**, APC/C ubiquitylation assay with radiolabeled full-length ySecurin, comparing wild-type APC/C^{Cdh1} and APC/C^{Cdh1} with a GFP tag on Apc1. **c**, Comparison of activities with APC/C^{Cdh1}, using either wild-type Cdh1 or Cdh1 with the N-terminal 2XStrep-Tag II. **d**, Comparison of activities with APC/C^{Cdh1} and the Hsl1 fragment (667-872) with and without the C-terminal Halo tag. **e**, Binding of radiolabeled Hsl1 fragments to yeast APC/C^{Cdh1} immobilized on magnetic beads. First lane (Inp, input) indicates the amount of labeled protein added to the beads prior to washing, second lane (+) indicates amount bound, and third lane (-) indicates background binding in absence of APC/C^{Cdh1}. **f**, Ubiquitylation of Halo-tagged Hsl1 fragment with APC/C^{Cdh1} and APC/C^{Cdc20}. Experiments were performed once (**b**, **c**) or twice (**d**-**f**). Source Data are provided as a Source Data file.

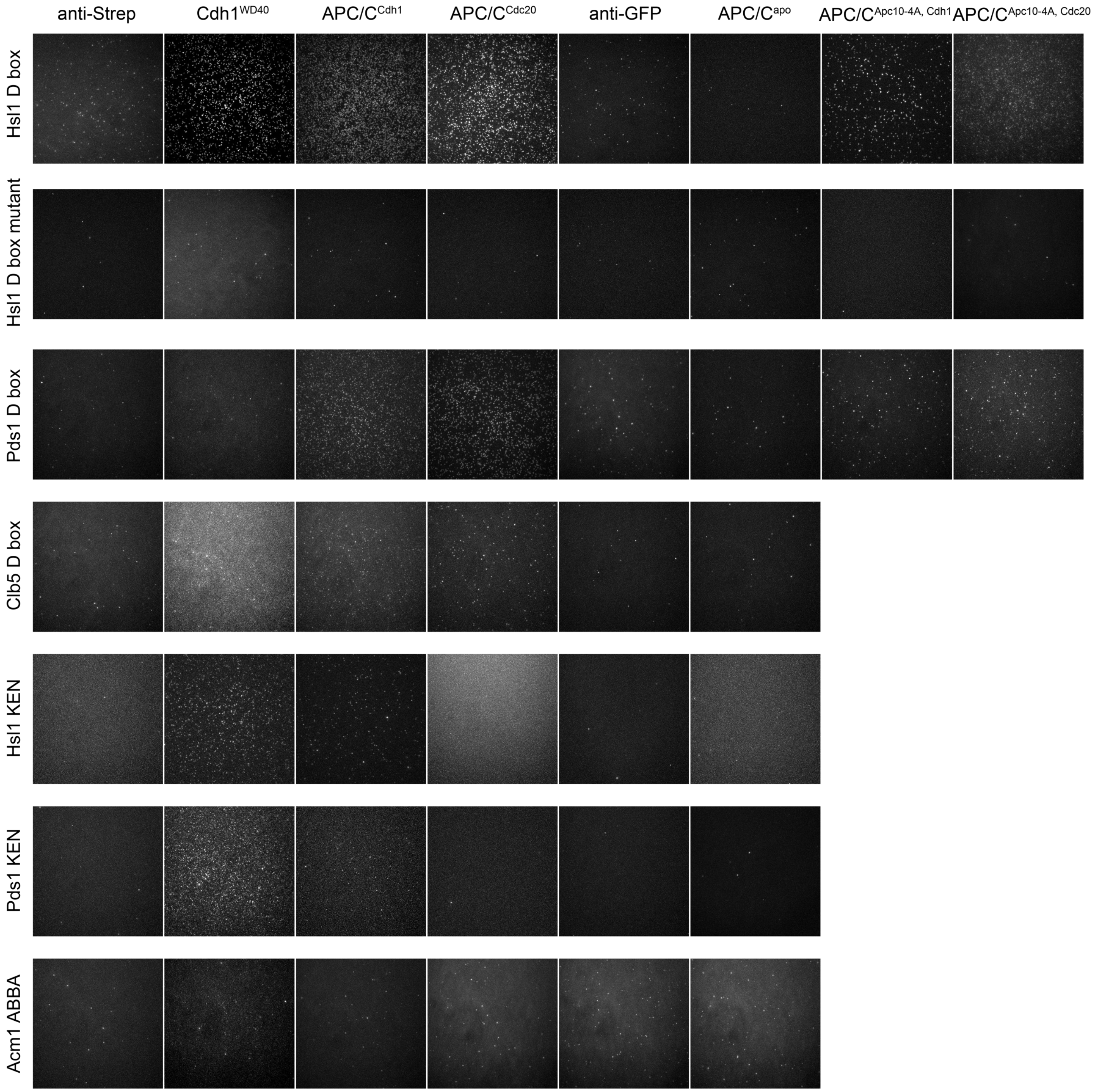

Supplementary Fig. 3: Yeast degron peptide binding in various conditions
Maximum intensity projections of 500 frames from videos of Cy5-labeled degron peptides (labeled on left) binding to various immobilized binding proteins (labeled at top). Images labeled 'anti-Strep' are background controls for binding to APC/C-activator complexes; images labeled 'anti-GFP' are background controls for binding to GFP-tagged APC/C^{apo} and Cdh1^{WD40}.

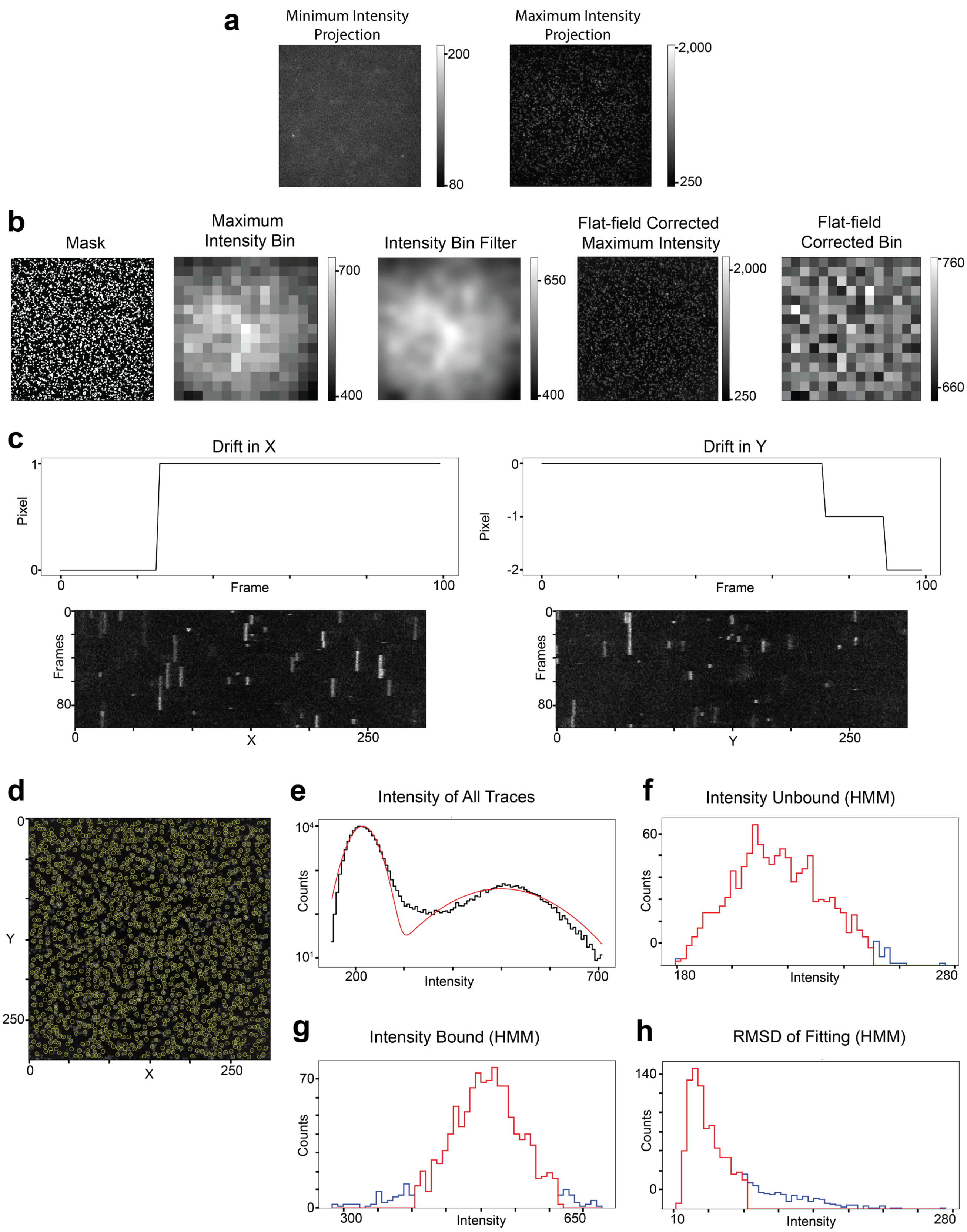

Supplementary Fig. 4: Steps in the SMOR analysis process

a, As a first step in the analysis, the code produces figures for maximum and minimum intensity projections (Z-stack) of the movie being analyzed. **b**, A mask is created by a binary filter to find the pixels with bright

spots. The mean intensity within the mask is calculated for each 20x20 pixel bin, and a Gaussian filter is applied to create the intensity bin filter. Flat-field correction is performed by normalizing the maximum intensity projection by the filter. As described in the main text, the process of flatfield correction produces several figures for the user to track the process (see Fig. 3b). **c**, Drift correction produces a plot to show the number of pixels of drift in both x and y axes as well as a kymograph in both x and y of the corrected movie for the user to determine if the drift has been adequately corrected. Each binding event produces a straight line in the kymograph, and drift results in a 1-2-pixel shift in all lines. **d**, The code initially identifies (x,y) coordinates where binding occurs in the movie by using a peak-finding algorithm and creates this peak identification plot. **e**, A histogram of minimum and maximum intensities along the entire trace (in time or z) at each (x,y) coordinate where a binding event was identified. Red line indicates double Gaussian fit of the data. **f**, Histogram of unbound intensities after HMM fitting. **g**, Histogram of bound intensities after HMM fitting. **h,** Plot of root mean squared deviation (RMSD) between the experimental trace and the HMM trace fitting among all (x,y) coordinates. For panels **f**-**h**, red indicates selected data (≤2 SD from the median) and blue indicates rejected data.

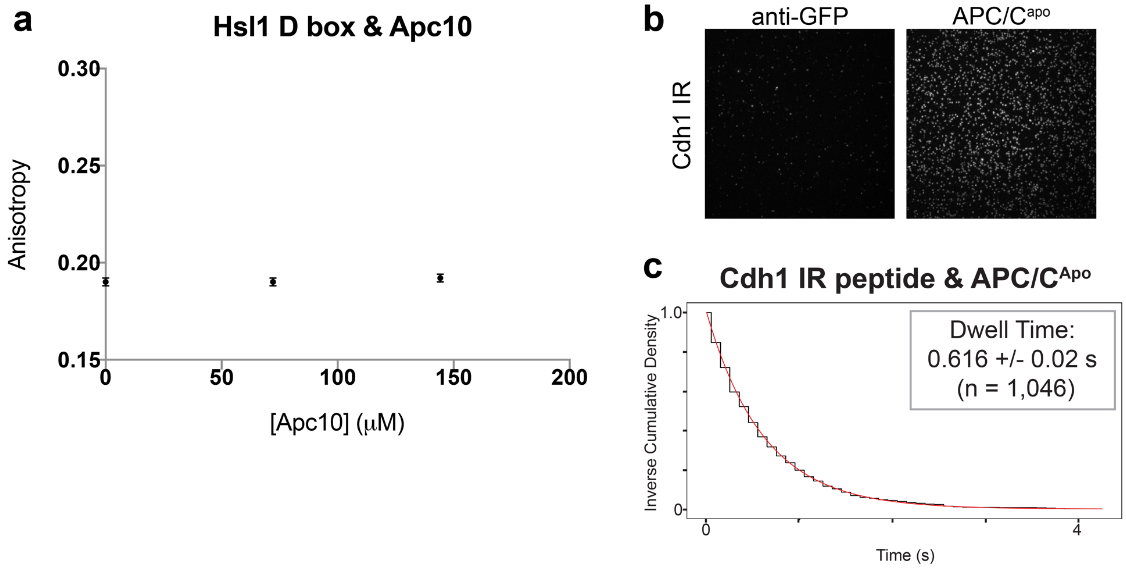

Supplementary Fig. 5: Control experiments with Apc10 and APC/C^{apo}
a, Fluorescence anisotropy analysis of 10 nM Cy-5 labeled Hsl1 D box peptide incubated with up to 144.3 µM purified yeast Apc10. Data points represent mean ± SE (n = 10 reads per reaction). Source data are provided as a Source Data file. **b**, Maximum intensity projection of 500 frames from a video of Cy5-labeled yeast Cdh1 C-terminal IR peptide ((Cy5)-SLIFDAFNQIR) with anti-GFP antibody to check for background binding and immobilized APC/C^{apo} to look at specific binding. **c**, Dwell time distribution from SMOR analysis of a representative movie of the Cdh1 IR peptide binding to APC/C^{Apo} immobilized at the surface.

a

Hsl1 D box	EQKPKRAALSDITNSFNKMN-K(Cy5)
Hsl1 D box mutant	EQKPKAAAASDITASFNKMN-K(Cy5)
AurKB D box	HNPSERLPLAQVSAHPWVRAN-K(Cy5)
Hsl1 KEN	GVSTNKENEGPEYPTKIE-K(Cy5)
hSecurin KEN	LIYVDKENGEPGTR-K(Cy5)

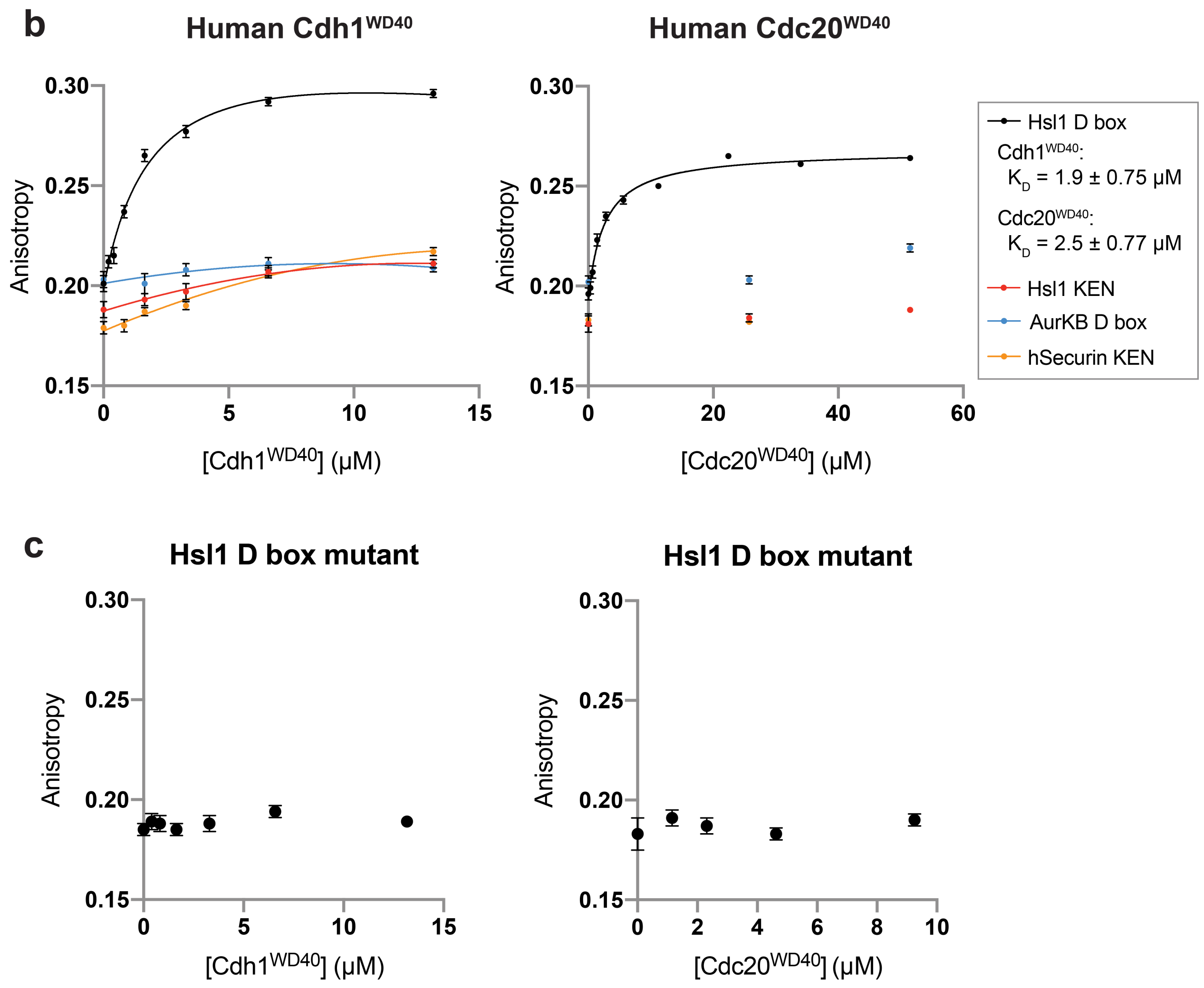

Supplementary Fig. 6: Anisotropy with human activator WD40
a, List of Cy5-labeled degron peptides tested in anisotropy experiments in **b**, **c**. **b**, Fluorescence anisotropy analysis of 10 nM Cy-5 labeled peptides binding to hCdh1^{WD40} or hCdc20^{WD40}. Data points represent mean ± SE (n = 10 reads per reaction). Inset indicates K_D values with estimated standard error calculated by GraphPad Prism. **c**, Analysis of mutant Hsl1 D box peptide with hCdh1^{WD40} or hCdc20^{WD40}. Data points represent mean ± SE (n = 10 reads per reaction). Source data are provided as a Source Data file.

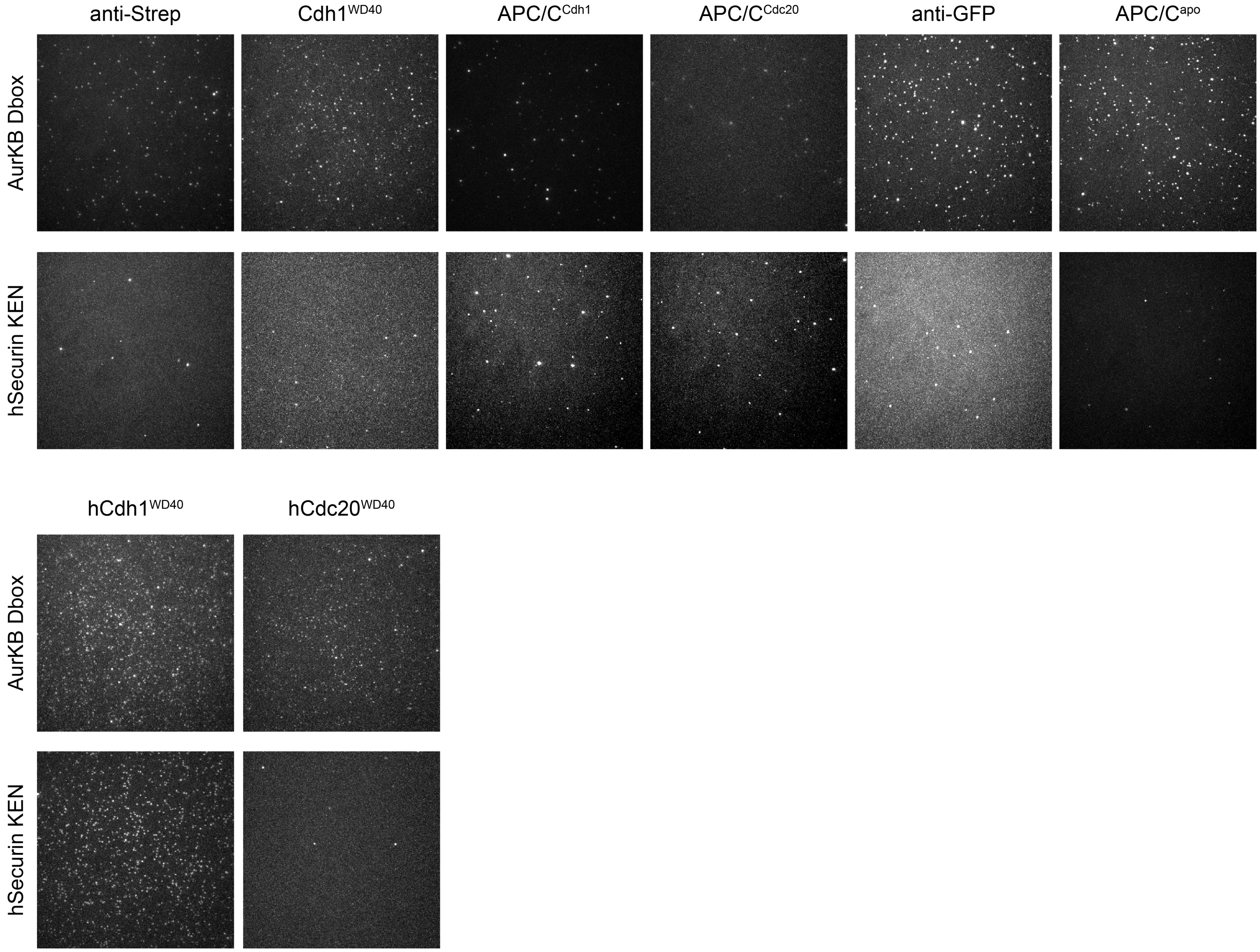

Supplementary Fig. 7: Human degron peptide binding in various conditions
Maximum intensity projections of 500 frames from videos of Cy5-labeled human degron peptides (labeled on left) binding to proteins immobilized at the surface (labeled at top): yeast APC/C and activators in top panels; human activator WD40 domains in bottom panels. Images labeled ‘anti-Strep’ are background controls for binding to APC/C-activator complexes; images labeled ‘anti-GFP’ are background controls for binding to GFP-tagged APC/C^{apo} and GFP-tagged activator WD40 domains.

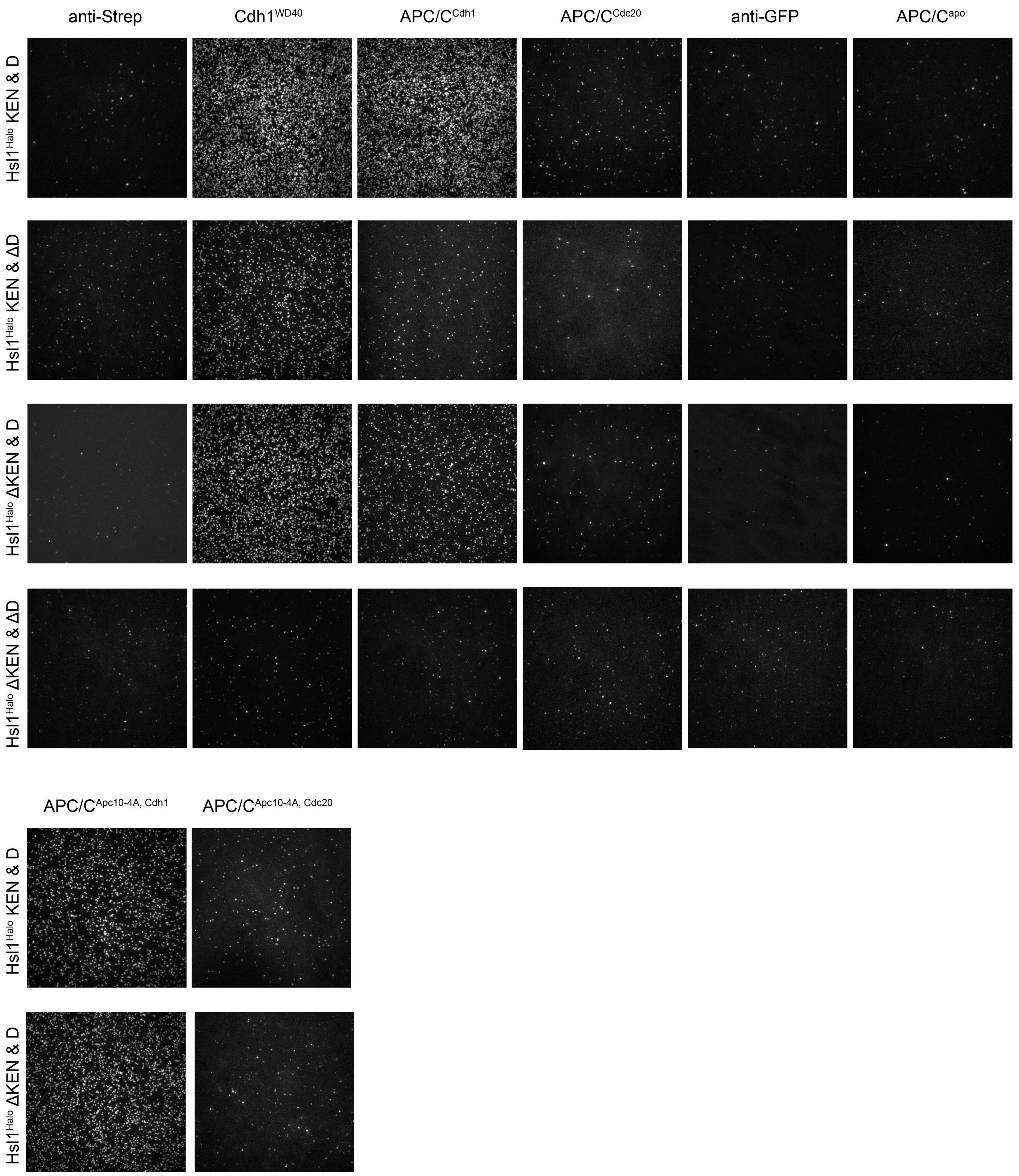

Supplementary Fig. 8: Hsl1^{Halo} binding signal in various conditions

Maximum intensity projections of 500 frames from videos of JF549-labeled wild-type and mutant Hsl1^{Halo} (labeled at left) binding to proteins immobilized at the surface (labeled at top). Images labeled 'anti-Strep' are background controls for binding to APC/C-activator complexes; images labeled 'anti-GFP' are background controls for binding to GFP-tagged APC/C^{apo} and Cdh1^{WD40}.

Supplementary Table 1: List of representative movies analyzed in this study

Yeast APC/C and Activators

Substrate	Glass Coverage	File Name	Exposure (ms)	Interval	Frames	Dwell Time (s)
Hsl1 D box	APC/C-Apo	25May2018_antieGFP_NHY13_peptide2-1nM_withPCAPC/CD_32_500count_0sinterval_1	32	0	500	no binding
	APC/C-Cdc20	12Nov2019_antistrep_APC/CCdc20_NHP2_1nM_1000frames_0sinterval_100msexp_1 X	100	0	1000	0.412 +/- 0.007 [s] (N = 6360)
	APC/C-Cdc20	13Dec2019_antistrep_APC/C-Cdc20_NHP2_1nM_1000frames_0ms_100msexp_R_2 X	100	0	1000	0.690 +/- 0.006 [s] (N = 20479)
	APC/C-Cdh1	3Jun2019_antistrep_NHY13+72_100pMNHP2_5s_100frames_100ms_1 X	100	5s	100	35.251 +/- 1.111 [s] (N = 858)
	APC/C-Cdh1	30Nov2018_antiSTREP_NHY13+Cdh1akapNH72_NHP2_100pM_300frames_5sec_32ms_R1_1 _A	32	5s	300	40.372 +/- 0.554 [s] (N = 6148)
	Cdh1 WD40	19April2019_antiSTREP_pNH148_NHP2_1000frames_100ms)R_1 X	100	0	1000	0.360 +/- 0.005 [s] (N = 4949)
	Cdh1 WD40	24Jan2019_antiStrep_pNH148_250pMNHP2_R_1000frames_100ms_1 X	100	0	1000	0.414 +/- 0.004 [s] (N = 7965)
	Apc10-4A-Cdc20	17Dec2019_antistrep_Apc10-4A+Cdc20_NHP2_1nM_32msexp_1000frames_0sinterval_1	32	0	1000	0.152 +/- 0.002 [s] (N = 7601)
	Apc10-4A-Cdc20	9July2020_antiStrep_DOMApc10-4A-pNH74_NHP2_1nM__32msexp_0msint_1000frames_4	32	0	1000	0.103 +/- 0.003 [s] (N = 1184)
	Apc10-4A-Cdh1	18Dec2019_antistrep_Apc10-4A+Cdh1_NHP2_100pM_100msexp_1000frames_0sinterval_1	100	0	1000	0.344 +/- 0.010 [s] (N = 1494)
	Apc10-4A-Cdh1	20Dec2019_antistrep_Apc10-4A-Cdh1_NHP2_1nM__100msexp_500frames_0sinterval_2	100	0	500	0.375 +/- 0.013 [s] (N = 908)
Hsl1 D box mutant	APC/C-Apo	17Dec2019_antieGFP_NHY13apo_NHP3_1nM_100sexp_500frames_0sinterval_1	100	0	500	no binding
	APC/C-Cdc20	13Dec2019_antistrep_APC/C-Cdc20_NHP3_1nM_500frames_0ms_100msexp_1 X	100	0	500	no binding
	APC/C-Cdc20	13Dec2019_antistrep_APC/C-Cdc20_NHP3_1nM_500frames_0ms_100msexp_G_1	100	0	500	no binding
	APC/C-Cdh1	17Dec2019_antistrep_NHY13+Cdh1_NHP3_1nM_100sexp_500frames_0sinterval_2	100	0	500	no binding
	Cdh1 WD40	1Nov2019_antistrep_pNH148_NHP3_1nM_1000frames_0sinterval_100msexp_1 X	100	0	1000	no binding
	Cdh1 WD40	13Dec2019_antistrep_pNH148_NHP3_1nM_500frames_0ms_100msexp_1 X	100	0	500	no binding
	Apc10-4A-Cdc20	27Jan2021_Apc10-4A_pNH74_NHP3(1nM)_100msexp_0msint_500frames_1 X	100	0	500	no binding
	Apc10-4A-Cdh1	25July2020_antistrep_Apc10-4A-pNH72_NHP3(1nM)__100msexp_0sint_500frames_1	100	0	500	no binding
ySecurin D box	APC/C-Apo	19July2020_antieGFP_APC/Capo_NHP4(1nM)__100msexp_0sint_500frames_1	100	0	500	no binding
	APC/C-Cdc20	27Jan2021_NHY13_pNH74_NHP4(1nM)_100msexp_500msint_400frames_3 X	100	500ms	1000	1.859 +/- 0.041 [s] (N = 1982)
	APC/C-Cdc20	30Sept2019_antistrep_APC/C-Cdc20_1nMNHP4_100msexp_500msin_400frames_2 X	100	500ms	400	1.870 +/- 0.035 [s] (N = 2747)
	APC/C-Cdh1	25Sept2019_antistrep_APC/C-Cdh1_1nMNHP4_32msexp_0in_1000frames_2 X	32	0	1000	0.132 +/- 0.002 [s] (N = 6853)
	APC/C-Cdh1	27Feb2019_antiSTREP_NHY13+pNH72_500pMNHP4_1000frames_0sec_interval_32ms_2 X	32	0	1000	0.121 +/- 0.006 [s] (N = 259)
	Cdh1 WD40	30Oct2019_antistrep_pNH148_NHP4_1nM_1000frames_0msinterval_32msexp_1 X	32	0	1000	single frame
	Cdh1 WD40	1Nov2019_antistrep_pNH148_NHP4_1nM_1000frames_0sinterval_100msexp_1 X	100	0	1000	single frame
	Apc10-4A-Cdc20	26Nov2019_antiStrep_DOMApc10-4A-pNH74_NHP4_1nM_1000frames_0sinterval_100msexp_2_X	100	0	1000	single frame
	Apc10-4A-Cdc20	17Dec2019_antistrep_Apc10-4A+Cdc20_NHP4_1nM_32_1000frames_0sinterval_1	32	0	1000	single frame
	Apc10-4A-Cdh1	17Dec2019_antistrep_Apc10-4A+Cdh1_NHP4_1nM_32msexp_1000frames_0sinterval_1	32	0	1000	single frame
	Apc10-4A-Cdh1	26Nov2019_antiStrep_DOMApc10-4A-pNH72_NHP4_1nM_1000frames_0interval_100msexp_2_X	100	0	1000	single frame
Hsl1 KEN	APC/C-Apo	19July2020_antieGFP_APC/Capo_NHP5(1nM)__100msexp_0sint_500frames_1	100	0	500	no binding
	APC/C-Cdc20	29JAN2019_antiStrep_NHY13_pNH74_500pMNHP5_R_100ms_0sinterval_1000frames_2	100	0	1000	no binding
	APC/C-Cdc20	30Sept2019_antistrep_APC/C-Cdc20_1nMNHP5_100msexp_0msin_1000frames_1 X no binding	100	0	1000	no binding
	APC/C-Cdh1	22Jan2021_NHY13+pNH72_NHP5(1nM)_32msexp_0msint_1000frames_R_1	32	0	1000	0.173 +/- 0.005 [s] (N = 1291)
	APC/C-Cdh1	25Sept2019_antistrep_APC/C-Cdh1_1nMNHP5_32msexp_0in_1000frames_2 X	32	0	1000	0.185 +/- 0.004 [s] (N = 1764)
	Cdh1 WD40	26August2019_antistrep_pNH148_NHP5_1nM_regular_1000frames_32ms_1 X	32	0	1000	0.164 +/- 0.002 [s] (N = 9562)
	Cdh1 WD40	19April2019_antiSTREP_pNH148_NHP5at1nM_1000frames_32ms_R_1 X	32	0	1000	0.166 +/- 0.003 [s] (N = 3595)
ySecurin KEN	APC/C-Apo	19July2020_antieGFP_APC/Capo_NHP7(1nM)__100msexp_0sint_500frames_1	100	0	500	no binding
	APC/C-Cdc20	19April2019_antiSTREP_NHY13_Cdc20_NHP7at1nM_1000frames_32ms_0sinterval_1 no binding	32	0	1000	no binding
	APC/C-Cdc20	30Sept2019_antistrep_APC/C-Cdc20_1nMNHP7_100msexp_0msin_1000frames_1 X no binding	100	0	1000	no binding
	APC/C-Cdh1	19April2019_antiSTREP_NHY13_Cdh1_NHP7at1nM_1000frames_32ms_0sinterval_3	32	0	1000	single frame
	APC/C-Cdh1	25Sept2019_antistrep_APC/C-Cdh1_1nMNHP7_32msexp_0in_1000frames_1 X	32	0	1000	single frame
	Cdh1 WD40	19April2019_antiSTREP_pNH148_NHP7at1nM_1000frames_32ms_0sinterval_R_1	32	0	1000	single frame
	Cdh1 WD40	1Nov2019_antistrep_pNH148_NHP7_1nM_1000frames_0sinterval_32msexp_1	32	0	1000	single frame
Acm1 ABBA	APC/C-Apo	12Nov2019_antieGFP_APC/Capo_NHP11_1nM_1000frames_0sinterval_100msexp_1	100	0	1000	no binding
	APC/C-Cdc20	27Nov2019_antistrep_NHY13-Cdc20_NHP11_1nM_500frames_0msinterval_100msexp_1 X	100	0	500	no binding
	APC/C-Cdc20	12Nov2019_antistrep_APC/CCdc20_NHP11_1nM_500frames_0sinterval_100msexp_1 X	100	0	500	no binding
	APC/C-Cdh1	27Nov2019_antistrep_NHY13-Cdh1_NHP11_1nM_500frames_0msinterval_100msexp_1 X	100	0	500	no binding
	APC/C-Cdh1	12Nov2019_antistrep_APC/CCdh1_NHP11_1nM_1000frames_0msinterval_100msexp_1 X	100	0	1000	no binding
	Cdh1 WD40	26August2019_antistrep_pNH148_NHP11_1nM_regular_1000frames_32ms_1 x	32	0	1000	single frame
	Cdh1 WD40	13Dec2019_antistrep_pNH148_NHP11_1nM_1000frames_0ms_32msexp_1	32	0	1000	single frame
Clb5 D box	APC/C-Apo	12Nov2019_antieGFP_APC/Capo_NHP14_1nM_1000frames_0sinterval_100msexp_1 X	100	0	1000	no binding
	APC/C-Cdc20	12Nov2019_antistrep_APC/CCdc20_NHP14_1nM_1000frames_0msinterval_32msexp_1 X	32	0	1000	0.179 +/- 0.008 [s] (N = 550)
	APC/C-Cdc20	13Dec2019_antistrep_APC/C-Cdc20_NHP14_1nM_1000frames_0ms_32msexp_2 X	32	0	1000	0.207 +/- 0.008 [s] (N = 776)
	APC/C-Cdh1	11Dec2019_antistrep_APC/C-Cdh1_NHP14_1nM_1000frames_0ms_60msexp_100gain_1	60	0	1000	0.146 +/- 0.007 [s] (N = 358)
	APC/C-Cdh1	2July2020_antiStrep_APC/CCdh1_NHP14_1nM_32msexp_0msint_1000frames_4	32	0	1000	0.159 +/- 0.004 [s] (N = 1467)
	Cdh1 WD40	30Oct2019_antistrep_pNH148_NHP14_1nM_1000frames_0msinterval_32msexp_1_X	32	0	1000	single frame
	Cdh1 WD40	11Dec2019_antistrep_pNH148_NHP14_1nM_2000frames_0ms_32msexp_100gain_1	32	0	2000	single frame
AurKB D box	APC/C-Apo	11May2021_anteGFP_APC/Capo_NHP17(1nM)_100msexp_0sint_500frames_1	100	0	500	no binding
	APC/C-Cdc20	25Jan2021_NHY13_pNH74_NHP17(1nM)_100msexp_0msint_500frames_1	100	0	500	no binding
	APC/C-Cdc20	28April2021_antisrep_NHY13+pNH74_NHP17(1nM)_100msexp_0sint_500frames_1 X	100	0	500	no binding
	APC/C-Cdh1	22Jan2021_NHY13+pNH72_NHP17(1nM)_100msexp_0msint_1000frames_R_1	100	0	1000	no binding
	APC/C-Cdh1	28April2021_antisrep_NHY13+pNH72_NHP17(1nM)_100msexp_0sint_500frames_1_X	100	0	500	no binding
	Cdh1 WD40	15Oct2020_pNH148_NHP17(1nM)_100msexp_0msinterval_1000frames_1	100	0	1000	single frame
	Cdh1 WD40	11Oct2020_pNH148_NHP17(1nM)_100msexp_0msinterval_1000frames_1 X	100	0	1000	single frame
hSecurin KEN	APC/C-Apo	11May2021_anteGFP_APC/Capo_NHP19(1nM)_100msexp_0sint_500frames_1	100	0	500	no binding
	APC/C-Cdc20	28April2021_antisrep_NHY13+pNH74_NHP19(1nM)_100msexp_0sint_500frames_1_X	100	0	500	no binding
	APC/C-Cdc20	1June2021_antisrep_NH13-Cdc20_NHP19(1nM)_100msexp_0msint_2000frames_1	100	0	500	no binding
	APC/C-Cdh1	28April2021_antisrep_NHY13+pNH72_NHP19(1nM)_100msexp_0sint_500frames_1_X	100	0	500	no binding
	APC/C-Cdh1	1Jun2021_antistrep_NH13-Cdh1_NHP19(1nM)_100msexp_0msint_1000frames_1	100	0	500	no binding
	Cdh1 WD40	11Oct2020_pNH148_NHP19(1nM)_32msexp_0msinterval_1000frames_2 X	32	0	1000	no binding
	Cdh1 WD40	15Oct2020_pNH148_NHP19(1nM)_100msexp_0msinterval_1000frames_1	100	0	1000	no binding
Hsl1^{Halo} KEN & D	APC/C-Apo	19Nov2019_antieGFP_APC/Capo_pNH85_1000frames_0sinterval_100msexp_1 X	100	0	1000	no binding
	APC/C-Cdc20	9July2020_antiStrep_NHY13-pNH74_pNH85(1-2)__100msexp_0sint_500frames_1	100	0	500	0.518 +/- 0.032 [s] (N = 351)
	APC/C-Cdc20	20Dec2019_antistrep_NHY13+Cdc20_pNH85undiluted_100msexp_500frames_0sinterval_2_X	100	0	500	0.396 +/- 0.027 [s] (N = 285)
	APC/C-Cdh1	10Oct2018_antiStrep_APC/C_pNH72_pNH85-PCAPC/CD_60frames_1min_1_A	100	1min	40	321.161 +/- 14.186 [s] (N = 577)
	APC/C-Cdh1	19Nov2019_antiStrep_APC/C-Cdh1_pNH85_1-10dilution_60frames_1mininterval_100msexp_1 X	100	1min	60	268.262 +/- 10.979 [s] (N = 794)
	Cdh1 WD40	3Feb2019_antiStrep_pNH148Cdh1wd40egfp_pNH85_100frames_10secinterval_100msexposure_2 A	100	10s	100	73.520 +/- 3.075 [s] (N = 777)
	Cdh1 WD40	19Nov2019_antiStrep_pNH148_pNH85_1-100dilution_100frames_10sinterval_100msexp_2 X	100	10s	100	56.447 +/- 2.072 [s] (N = 926)
	Apc10-4A-Cdc20	18Dec2019_antistrep_Apc10-4ACdc20_pNH85higherconc_100msexp_1000frames_0sinterval_1	100	0	1000	0.449 +/- 0.014 [s] (N = 1470)
	Apc10-4A-Cdc20	9July2020_antiStrep_DOMApc10-4A-pNH74_pNH85(1-2)__100msexp_0msint_1000frames_1	100	0	218	0.372 +/- 0.027 [s] (N = 264)
	Apc10-4A-Cdh1	20Dec2019_antistrep_Apc10-4A-Cdh1_pNH85(1-100dilution__100msexp_500frames_10sinterval_1_X	100	10s	100	42.881 +/- 2.489 [s] (N = 269)
	Apc10-4A-Cdh1	25July2020_antistrep_Apc10-4A-pNH72_pNH85(1-10dilution)__100msexp_10sint_100frames_1	100	10s	100	75.919 +/- 3.159 [s] (N = 664)
Hsl1^{Halo} KEN & ΔD	APC/C-Apo	2July2020_antiStrep_APC/Capo_pNH95(1-10)__100msexp_0msint_500frames_1	100	0	500	no binding
	APC/C-Cdc20	20Dec2019_antistrep_NHY13+Cdc20_pNH95(nodilution)_100msexp_500frames_0sinterval_1	100	0	500	no binding
	APC/C-Cdc20	25Jan2021_NHY13_pNH74_pNH95(1-2)_100msexp_0msint_500frames_1	100	0	500	no binding
	APC/C-Cdh1	18Dec2019_antistrep+NH13Cdh1_pNH95_higherconc_100msexp_1000frames_0sinterval_1	100	0	1000	0.357 +/- 0.017 [s] (N = 615)
	APC/C-Cdh1	2July2020_antiStrep_APC/CCdh1_pNH95highcon_100msexp_0sint_500frames_1	100	0	500	0.374 +/- 0.015 [s] (N = 868)
	Cdh1 WD40	20Dec2019_antistrep_pNH148_pNH95(nodilution)_100msexp_1000frames_0sinterval_1 X	100	0	1000	0.489 +/- 0.006 [s] (N = 7999)
	Cdh1 WD40	22Jan2021_pNH148_pNH95(1-2)_100msexp_0msint_1000frames_1	100	0	1000	0.421 +/- 0.008 [s] (N = 2960)
Hsl1^{Halo} ΔKEN & D	APC/C-Apo	20Dec2019_antieGFP_APC/Capo_pNH114_100msexp_500frames_0sinterval_1	100	0	500	no binding
	APC/C-Cdc20	20Dec2019_antistrep_NHY13+Cdc20_pNH114undiluted_100msexp_500frames_0sinterval_1	100	0	500	single frame
	APC/C-Cdc20	27Jan2021_NHY13_pNH74_pNH114(1-2)_100msexp_0sint_1000frames_2 X	100	0	1000	single frame
	APC/C-Cdh1	13March2019_antiSTREP_NHY13+pNH72-Cdh1_1-10pNH114_32ms_5sinterval_200frames_1 X	32	5s	200	21.981 +/- 1.208 [s] (N = 385)
	APC/C-Cdh1	27Jan2021_NHY13_pNH72_pNH114(1-10)_100msexp_5sint_200frames_1 X	100	5s	200	41.594 +/- 2.105 [s] (N = 403)
	Cdh1 WD40	27Jan2021_pNH148_pNH114(1-10)_100msexp_500msint_400frames_1 X	100	500ms	400	1.724 +/- 0.066 [s] (N = 869)
	Cdh1 WD40	20Dec2019_antistrep_pNH148_pNH114_100msexp_400frames_500msinterval_1	100	500ms	400	3.095 +/- 0.065 [s] (N = 2857)
	Apc10-4A-Cdc20	18Dec2019_antistrep_Apc10-4ACdc20_pNH114higherconc_100msexp_1000frames_0sinterval_1	100	0	1000	single frame
	Apc10-4A-Cdc20	27Jan2021_Apc10-4A_pNH74_pNH114(1-2)_100msexp_0sint_1000frames_1 X	100	0	1000	single frame
	Apc10-4A-Cdh1	18Dec2019_antistrep_0930Cdh1_pNH114_100sexp_300frames_500msinterval_1	100	500ms	300	3.592 +/- 0.121 [s] (N = 1234)
	Apc10-4A-Cdh1	25July2020_antistrep_Apc10-4A-pNH72_pNH114(1-2dilution)__100msexp_500msint_500frames_1	100	500ms	500	3.308 +/- 0.060 [s] (N = 4032)
Hsl1^{Halo} ΔKEN & ΔD	APC/C-Apo	29January2019_eGFP_APC/Capo_pNH162_1000frames_32ms_0interval_R_1	32	0	1000	no binding
	APC/C-Cdc20	29January2019_antiStrep_NHY13+pNH74_washedagain_fullconcpNH162added_1000frames_32ms_0interval_R_1 X	32	0	1000	no binding
	APC/C-Cdc20	20Dec2019_antistrep_NHY13+Cdc20_pNH162undiluted_100msexp_500frames_0sinterval_1	100	0	500	no binding
	APC/C-Cdh1	18Dec2019_antistrep_NHY13Cdh1_pNH162_32sexp_1000frames_0sinterval_1	32	0	1000	no binding
	APC/C-Cdh1	29January2019_antiStrep_NHY13_pNH72_pNH162_R_100ms_0sinterval_1000frames_2	100	0	1000	no binding
	Cdh1 WD40	20Dec2019_antistrep_pNH148_pNH162_100msexp_500frames_0msinterval_1	100	0	500	no binding
	Cdh1 WD40	3Feb2019_antiStrep_pNH148Cdh1wd40egfp_pNH162_1000frames_0sinterval_100ms_R_1 X	100	0	1000	no binding
Cdh1 IR	APC/C-Apo	14September2018_antieGFP_NHY13_NHP6_R_100ms_1000frames_1	100	0	1000	0.616 +/- 0.019 [s] (N = 1046)
	APC/C-Apo	7Sept2021_antieGFP_nhy13_NHP6(1nM)_100ms_0sint_1000frames_1	100	0	1000	0.557 +/- 0.011 [s] (N = 2368)

Human Activators

Substrate	Glass Coverage	File Name	Exposure (ms)	Interval	Frames	Dwell Time (s)
Hsl1 D box	Cdc20 WD40	3Oct2020_pNH190_NHP2(1nM)_100msexp_0sinterval_500frames_1 X	100	0	500	1.077 +/- 0.016 [s] (N = 4083)
	Cdc20 WD40	25Oct2020antiStrep_pNH190_NHP2(100pM)_100msexp_0msinterval_1000frames_2	100	0	1000	1.084 +/- 0.015 [s] (N = 4669)
	Cdh1 WD40	10June2020_antistrep_pNH170_smAPC/Cbufer_50pMNHP2_100msexp_1sint_200frames_1 X	100	1s	200	5.039 +/- 0.073 [s] (N = 4737)
	Cdh1 WD40	28April2021antisrep_pNH170_NHP2(100pM)_100msexp_1smsint_200frames_1_XA	100	1s	200	4.515 +/- 0.095 [s] (N = 2272)
Hsl1 D box mutant	Cdc20 WD40	3Oct2020_pNH190_NMHP3(1nM)_100msexp_0sinterval_500frames_R_1 x	100	0	500	no binding
	Cdh1 WD40	10June2020_antistrep_pNH170_smAPC/Cbuffer_1nMNHP3_100msexp_0sint_200frames_1	100	0	200	no binding
ySecurin D box	Cdc20 WD40	3Nov2020_antiStrep__pNH190_NHP4(1nM)_32msexp_0msinterval_1000frames_1 X	32	0	500	single frame
	Cdc20 WD40	28April2021_antisrep_pNH190_NHP4(1nM)_100msexp_0sint_500frames_1_X	100	0	500	single frame
	Cdh1 WD40	16August2020_antisrep_pNH170_NHP4(1nM)__32msexp_0sint_500frames_1	32	0	500	single frame
	Cdh1 WD40	9July2020_antiStrep_pNH170_NHP4at1nM__32msexp_0msint_1000frames_1	32	0	1000	single frame
Hsl1 KEN	Cdc20 WD40	9Dec2020_antistrep_pNH190_NHP5_32msexp_0sinterval_1000frames_WITHpcAPC/Cd_1	32	0	1000	single frame
	Cdc20 WD40	18Nov2020_antiStrep_pNH190_NHP5(1nM)_32msexp_0sinterval_1000framesa_1 X	32	0	1000	single frame
	Cdh1 WD40	20June2020_antiStrep_pNH170_50pMNHP5_100msexp_250msint_500frames_1	100	250ms	500	1.910 +/- 0.054 [s] (N = 1091)
	Cdh1 WD40	16August2020_antistrep_pNH170_NHP5(1nM)__100msexp_250msint_500frames_1	100	250ms	500	1.483 +/- 0.021 [s] (N = 4893)
ySecurin KEN	Cdc20 WD40	3Nov2020_antiStrep__pNH190_NHP7(1nM)_32msexp_0msinterval_1000frames_1 XD	32	0	1000	single frame
	Cdc20 WD40	9Dec2020_antistrep_pNH190_NHP7_32msexp_0sinterval_1000frames_WITHpcAPC/Cd_1	32	0	1000	single frame
	Cdh1 WD40	25July2020_antistrep_pNH170_NHP7(500pM)__100msexp_0sint_500frames_1	100	0	500	0.869 +/- 0.013 [s] (N = 4509)
	Cdh1 WD40	16August2020_antistrep_pNH170_NHP7(1nM)__100msexp_0msint_500frames_4	100	0	500	0.929 +/- 0.013 [s] (N = 4773)
Acm1 ABBA	Cdc20 WD40	3Nov2020_antiStrep__pNH190_NHP11(1nM)_100msexp_0msinterval_1000frames_1 X	100	0	1000	no binding
	Cdc20 WD40	28April2021_antisrep_pNH190_NHP11(1nM)_100msexp_0sint_500frames_1_X	100	0	500	no binding
	Cdh1 WD40	9July2020_antiStrep_pNH170_NHP11at1nM__100msexp_0msint_1000frames_1	100	0	1000	no binding
	Cdh1 WD40	28April2021_antisrep_pNH170_NHP11(1nM)_100msexp_0sint_500frames_1_X	100	0	500	no binding
Clb5 D box	Cdc20 WD40	11May2021_antistrep_pNH190_NHP14(100pM)_20msexp_0sint_1000frames_smallerROI_1	20	0	1000	0.054 +/- 0.001 [s] (N = 2302) Not 3x shutter speed
	Cdc20 WD40	14May2021_antistrep_pNH190_NHP14(50pM)_32msexp_0sint_1000frames_3	32	0	1000	0.058 +/- 0.001 [s] (N = 2575) Not 3x shutter speed
	Cdh1 WD40	11May2021_antistrep_pNH170_NHP14(100pM)_32msexp_0sint_1000frames_1	32	0	1000	single frame
	Cdh1 WD40	28April2021_antisrep_pNH170_NHP14(1nM)_32msexp_0sint_1000frames_1	32	0	500	single frame
AurKB D box	Cdc20 WD40	3Oct2020_pNH190_NHP17(1NM)_32msexp_0sinterval_1000frames_1 X	32	0	1000	single frame
	Cdc20 WD40	11Oct2020_pNH190_NHP17(500pM)_32msexp_0msinterval_1000frames_1 X	32	0	1000	single frame
	Cdh1 WD40	9Sept2020_pNH170_NHP17(1nMl)_0sinterval_1000frames_50msexp_1	50	0	1000	0.202 +/- 0.003 [s] (N = 6572)
	Cdh1 WD40	25Oct2020_antiStrep__pNH170_NHP17(1nM)_32msexp_0msinterval_1000frames_1	32	0	1000	0.135 +/- 0.003 [s] (N = 2575)
hSecurin KEN	Cdc20 WD40	3Oct2020_pNH190_NHP19(100pM)_100msexp_0sinterval_500frames_1 X	100	0	500	no binding
	Cdc20 WD40	15Oct2020_pNH190_NHP190(1nM)_100msexp_0msinterval_500frames_1	100	0	500	no binding
	Cdh1 WD40	3Oct2020_pNH170_NHP19(100pM)_32msexp_0sinterval_1000frames_2 X	32	0	1000	0.171 +/- 0.004 [s] (N = 1390)
	Cdh1 WD40	25Oct2020_antiStrep__pNH170_NHP19(500pM)_32msexp_0msinterval_1000frames_1	32	0	1000	0.174 +/- 0.003 [s] (N = 3431)

Exposure = Shutter speed
Interval = interval between images; when 0, movie was taken continuously at the exposure rate

Supplementary Table 2: Yeast strains

NHY13	cdh1::LEU2<br>bar1::hisG<br>CDC16::CDC16-TAP-HIS3<br>APC1::APC1-GFP-CaUra<br>MATa mating type, W303 background
DOM1226	cdh1:: LEU2<br>bar1::hisG<br>CDC16::CDC16-TAP-HIS3
DOM0930	CDC16::CDC16-TAP-HIS3<br>doc1d::URA3<br>trp1::TRP1-pRS304-doc1-4A

Supplementary Table 3: Bacmid vectors for protein expression

pNH72	2xStrep-Tag II-yeast Cdh1
pNH74	2xStrep-Tag II-yeast Cdc20
pNH144	2xStrep-Tag II-yeast Cdh1 WD40
pNH148	2xStrep-Tag II-yeast Cdh1 WD40-GFP
pNH164	2xStrep-Tag II-human Cdh1 WD40
pNH170	2xStrep-Tag II-human Cdh1 WD40-GFP
pNH175	2xStrep-Tag II-yeast Apc10
pNH188	2xStrep-Tag II-human Cdc20 WD40
pNH190	2xStrep-Tag II-human Cdc20 WD40-GFP

Supplementary Table 4: Peptide sequences

NHP2	Hsl1 D box	EQKPKRAALSDITNSFNKMN-K(Cy5)
NHP3	Hsl1 D box mutant	EQKPKAAAASDITASFNKMN-K(Cy5)
NHP4	ySecurin (Pds1) D box	AQQQGRLPLAAKDNNRSKSFI-K(Cy5)
NHP5	Hsl1 KEN	GVSTNKENEGPEYPTKIE-K(Cy5)
NHP6	Cdh1 IR	(Cy5)-SLIFDAFNQIR
NHP7	ySecurin (Pds1) KEN	PANEDKENNIVYTG-K(Cy5)
NHP8	Unlabeled Hsl1 D box	EQKPKRAALSDITNSFNKMN
NHP9	Unlabeled Hsl1 KEN	GVSTNKENEGPEYPTKIE
NHP11	Acm1 ABBA	SKAAQFMLYEETAEERNI-K(Cy5)
NHP14	Clb5 D box	QDSKPRRALTDVPVNNNPLSQ-K(Cy5)
NHP17	AurKB D box	LPKATRKALGTVNRATEKSVK-K(Cy5)
NHP19	hSecurin(Pttg1) KEN	LIYVDKENGEPGTR-K(Cy5)

Supplementary Table 5: *In vitro* translation plasmids (contain T7 promoter)

pNH85	Hsl1$^{667-872}$-Halo-TEV-ZZ
pNH95	Hsl1$^{667-872}$-Halo-TEV-ZZ with R,L, & N in D box mutated
pNH114	Hsl1$^{667-872}$-Halo-TEV-ZZ with KEN mutated to AAA
pNH162	Hsl1$^{667-872}$-Halo-TEV-ZZ with KEN & D box mutations
pJK567	hSecurin (Pttg1)-TEV-ZZ
pME39	ySecurin(Pds1)-TEV-ZZ
pME60	Hsl1$^{667-872}$-TEV-ZZ

Supplementary Note 1: Detailed description of SMOR analysis pipeline

Fluorescent ligand in solution repeatedly binds and unbinds to its binding partner attached to the glass surface. Here, the dwell time during the surface-bound state is determined by the dissociation rate (k_off). When bound, the fluorescent molecule is excited by the TIRF illumination, emits fluorescence that is captured by the camera and appears as a bright spot in the time-lapse movie. In our setup, magnification is adjusted such that each isolated fluorescent spot occupies a ~3x3 pixel area in the movie. The main goal of the analysis is to extract the dissociation rate from each movie containing many such single molecule binding/unbinding (dwell) events. Here, we assume that all molecules are homogenous and follow the same kinetic rate. In order to achieve this goal, the program identifies the pixel locations where the binding/unbinding events take place, measures the intensity changes over time, identifies the transition between bound and unbound states, aggregates all the dwell events from many molecules at different locations, and calculates the mean dwell time from the dwell time distribution, which is an inverse of the dissociation rate (k_off). As part of the analysis, the algorithm identifies and excludes outliers (such as fluorescent junk or multiple molecules in proximity) and corrects the data (i.e. drift correction and flatfield correction) for better performance. Below are more details of the analysis pipeline, with instructions to allow subsequent users to run the code and adapt the algorithm for their unique experiment/binding data.

Before running the analysis, data needs to be organized in the following manner.

- Data folder
  - data1
    - movie1.tif
    - info.txt
  - data2
    - movie2.tif
    - info.txt

Each data folder must have a single movie file in a tiff file format and an info.txt file that contains the custom parameters for the analysis of each movie. Running a command `run-smor-analysis` starts reading all the movie files under the current working directory and analyzing each movie one by one. Once the analysis is successfully completed, the corresponding figures and a summary file (result.txt) is generated in each data. If an error occurs while analyzing one of the movies, a log file (error.txt) that describes the specific error messages is created in the corresponding data folder.

The analysis pipeline consists of the following steps.

1) Read the movie in a tiff format and the info.txt file.
2) Do flatfield correction.
3) Do drift correction.

4) Find fluorescent intensity peak locations from the maximum intensity Z-projection image.
5) Find the fluorescent spots with similar brightness.
6) For each fluorescent spot trace, apply two-state Hidden Markov Model (HMM) to identify the transition between bound and unbound states.
7) Collect the bound state dwell time from the HMM analysis and calculate the unbinding kinetics.
8) If completed successfully, save the figures and the result file. If not, save an error log file.
9) Move on to the next data folder and repeat.

Description of steps:

1) Read the movie and the info.txt file:

The code first reads the info.txt file that describes the specifics of the movie and the user-defined custom parameters for analysis. These custom parameters include basic information such as the time interval between frames, spot size in pixels, data filtering conditions, and more. A complete list of all parameters is described at the end. The code uses a python library Tiff file to read the movie in a tiff format. The pixel intensity is loaded in a 3D array (NumPy) where the 1st axis is frame while the 2nd and 3rd are the row and column of each frame image. Then, the image is cropped to 300 x 300 pixels at the center, where the intensity is normally brighter than the edges. If the user finds that the TIRF field is off center, the movie can be cropped to 300 x 300 pixels beforehand.

2) Flatfield correction:

This code uses the intensity distribution at bright spot locations across the field of view to judge whether to include or exclude those spots. Basically, we want to collect data with similar brightness and exclude outliers. If intensity at a spot is much brighter than other spots, it could be due to multiple spots in proximity or aggregates; hence, they are excluded from the analysis. If intensity is much darker, then it could be fluorescent junk, not valid signal. This approach is valid only if the illumination for the fluorescence signal is uniform across the field of view and the intensity differences is not simply due to non-uniform illumination. In reality, illumination is never completely uniform. In general, the central area is brighter than the outer area due to the Gaussian laser beam profile (Supplementary Fig. 4b). Moreover, higher order non-linear patterns might also exist due to aberrations or suboptimal alignment of the optics. Therefore, we used a flatfield correction algorithm to normalize each pixel intensity by the average intensity in the local area. We first make a mask using local binary filter (threshold_local method from skimage library) to identify the pixels where fluorescent spots are located (Supplementary Fig. 4b). Then, we obtain the mean intensity within the mask in each bin area (Supplementary Fig. 4b). If the bin size is too small, then it reveals a more detailed spatial pattern of illumination, but it becomes less accurate due to a smaller number of mask pixels residing in the bin average. A bin size that is too large yields the opposite effect. We found that a 20 x 20 pixel area works best with our data. If the density of spots is too low, one needs to increase the binning size, and vice versa. The locally averaged image is smoothened a bit using a gaussian filter to remove the sharp contrast between the neighboring bins (Supplementary Fig. 4b). The original image is normalized by the smoothened image to compensate for non-uniform illumination (Supplementary Fig. 4b). The flatfield-corrected image is once again binned and averaged to double check that the spatial pattern has less structure and looks random (Supplementary Fig. 4b). The user can allow or skip this correction by a custom parameter (flatfield_correct = true or false) as needed.

3) Drift correction:

The microscope sample slide can drift over time and the effect becomes more noticeable if the movie is taken for a long period of time or the microscope setup is less stable. This sample drift can cause problems in our analysis as we use a fixed pixel location to measure the fluorescent intensity changes over time. To solve this problem, the program detects the image drift throughout the frames over time and compensates the amount of drift for every frame. We use an open-source python library for image analysis (*imreg_dft*), using its 'translation' method to measure image drift[33]. This method compares two images (test and reference) and finds the translation of the test image in row and column directions as compared to the reference image, using spatial correlation between the two images. In other words, pattern matching with translation is used between the two images. We don't consider other transformations, such as rotation or magnification, as their effects are less significant than translation. For our analysis, the image at the mid

frame was used as a reference as it has the minimum pairwise frame differences with all the other frames; hence, it contains more overlapping spots between the two images. All the fluorescent spots are used as fiducial markers, but only part of them present in both images due to the stochastic nature of binding and unbinding processes. Nevertheless, the algorithm works well if there are sufficient overlapping spots in the two images. Afterwards, additional filters are applied to improve the robustness, since the translation algorithm alone can be affected by unwanted noise, such as bright fluorescent aggregates in some frames. We set a maximum allowed translation between two consecutive frames (default = 2 pixels), followed by a moving average for a lowpass filter, to avoid abrupt jumps between frames, which is more likely due to artifact rather than actual drift since the most dominant drift effect is at low frequency. Drift in both axes can be noted per movie as shown in Supplementary Fig. 4c. The user can allow or skip this correction with a custom parameter (drift_correct = true or false) as needed. The need for drift should be determined by the user based on their TIRF microscope. For our microscope, we applied it for acquisition intervals above 500 miliseconds.

4) Find fluorescent intensity peak locations from the maximum intensity Z-projection image:
After the two previous corrections, we are ready to find the pixel locations where fluorescent ligand binds and unbinds. We use the maximum intensity Z-projection image along the time axis to accumulate all the bright spots from all the frames in the movie. To find the potential binding sites, we use the peak_local_max method from the python library scikit-image, which returns the local maximum peaks from the image. We set a minimum distance between the closest spots allowed to avoid any interference between them. Current default min_distance value is set from the custom parameter (equivalent to spot_size in pixel), so that two peaks cannot be chosen within the area occupied by a single spot. The selected peaks are shown in Supplementary Fig. 4d in yellow circles. Subsequently, the average intensity within the spot_size pixel area around each peak is calculated for each frame to measure the intensity changes of the individual spots throughout the frames, and saved as peak_trace. This averaging filter is applied to sum all the signal from each spot around the peak. Some of the peaks are from true binding/unbinding processes, while others could be from inactive fluorescent junk or aggregates of multiple molecules, which will be filtered out in the subsequent steps.

5) Find the fluorescent spots with similar brightness:
With uniform illumination of fluorescent molecules or with flatfield correction, we can assume that all the fluorescent spots from single isolated fluorophores have similar brightness. Outliers such as very bright signal could be due to multimer and much darker signal could be fluorescent junk. Hence, we use their intensity distribution and cutoff criteria to filter out the outliers. We use both the minimum intensity as well as the maximum intensity distribution as shown in Fig. 3c to utilize the information from both bound and unbound states. The maximum (or minimum) intensity value from each peak trace is collected to form a histogram (Fig. 3c). As shown, the intensities are tightly distributed around the peaks. Spots where both their maximum and minimum intensities reside within multiples of standard deviation (SD) from their overall median intensities are selected as inliers (inliers in red and outliers in blue). The multiplication factor is determined by the custom parameters (intensity_min_cutoff and intensity_max_cutoff). For example, cutoff=2 yields the population within two standard deviations from the median intensity. In some experiments, we have observed multiple peaks in the intensity distributions, which indicates that the data includes a mixture of multiple groups. For example, one maximum intensity peak is from normal binding whereas another peak is from transient binding events yielding lower intensity or multimers with brighter intensity. To give the user more options in these scenarios, custom parameters (intensity_min_num, intensity_max_num) are used to set the number of groups or peaks in the intensity distributions, and the parameters (intensity_min_index, ntensity_max_index) are the index of the group we want to include in the analysis. The Gaussian Mixture model from scikit-learn python library was used for the classification of the groups. The selected traces (red) with three parameters (cutoff, num, index) from both min and max intensities are used in the following step to characterize the binding/unbinding kinetics.

6) For each intensity trace from the selected spots, apply two-state Hidden Markov Model (HMM) to identify the transition between bound and unbound states:
Supplementary Fig. 4e is the histogram of intensities along the entire traces of all selected spots. The distribution fits well with the sum of two Gaussian distributions (in red) with means at low and high

intensities, which agrees well with our two-states model. Each individual intensity trace shows the transition between unbound state at low intensity and bound state at high intensity, as shown in the example trace figures (Fig. 3d, e). Depending on the experimental parameters, including time interval per frame, total number of frames, and unbinding/binding kinetics, the number of binding/unbinding cycles changes. Fluorescence intensity fluctuates due to several factors, such as intrinsic blinking of the fluorophore, laser power fluctuation, or freely diffusing fluorophores in the background. Hence the true molecular state, either bound or unbound, is hidden under noise and we need to estimate them with a statistical model. A Hidden Markov model (HMM) is a statistical Markov model with hidden states, where the transition probability between states is determined by the current state[34]. We consider a two-state HMM as having only an unbound or single fluorophore-bound state. If the experimental data contains multiple binding states, such as dimers or trimers, the number of states in the HMM needs to be extended (and the code needs to be modified accordingly). For our analysis, we use a python library hmmlearn, and we specifically use the GaussianHMM model as we can assume Gaussian emissions for the intensity fluctuation. HMM uses the entire dataset and tweaks the parameters to maximize the likelihood of observing the experimental data. This means it is robust to the noise sources affecting the state determination, such as blinking or transient spikes, which is superior to other threshold-based methods. For more robust fitting, we use the initial condition of the model parameters from the experimental data, including fractions, means, and variances of the two states from the fitting in Supplementary Fig. 4e. Then, the individual trace was fitted into the model to predict the HMM parameters (means, variances, transition probability) as well as the states per frame. After the fitting of the entire traces, we apply additional filters with selection criteria to exclude outliers. Here, we use three criteria based on the mean intensity of unbound states (Supplementary Fig. 4f), bound states (Supplementary Fig. 4g), and the root-mean-squared-deviation (RMSD) between experimental trace and the trace fitting (Supplementary Fig. 4h). We use custom parameters (HMM_RMSD_cutoff, HMM_bound_cutoff, HMM_unbound_cutoff) to select the populations near the peaks within the cutoff multiples of their standard deviations. The traces satisfying all three criteria are finally chosen for the following kinetics analysis. In some experimental conditions, we observed multiple peaks in the bound state intensity distribution, which indicates that there are multiple bound states in the experimental data, such as dark intensity due to transient binding or brighter intensity due to multiple fluorophore binding, regardless of our two states HMM model. In order to give more options to the users in these cases, we use a GaussianMixture model with custom parameters (HMM_bound_num, HMM_bound_index) to select those populations.

7) Collect the bound state time from the HMM analysis and calculate the unbinding kinetics:
Some of the traces show multiple binding/unbinding events while others have only one or part of the signal (pre-existing or unfinished bound state). To simplify the analysis, we only collect complete bound state times that remain in the bound state from the beginning to the end. First, we collect all the bound state time (i.e. dwell time) from all of the traces. Subsequently, unwanted short or long events can be filtered out. In a short time period, there could be missing events during the sampling or contamination of transient binding events. In a long time period, there could be missing events due to photo bleaching or artifacts due to fluorophores being stuck to the surface. A custom parameter (frame_offset) can exclude short events by offsetting the value from all of the dwell times. Very long dwell outliers that are longer than 10-fold of the median dwell time are automatically excluded from the dwell time collection. Dwell time distribution can be assumed as an exponential distribution with a dissociation rate constant, and in this scenario, the maximum likelihood estimator of the dissociation constant is the inverse of the sample mean dwell time. We can check whether this assumption is valid by comparing the exponential function with the experimental data in the final dwell time histogram where the decay parameter from the experimental sample mean dwell time in red overlays the normalized experimental dwell time histogram in black (as seen in Fig. 4a). Our code represents the data as both a probability density function or an inverse cumulative density function. As expected, the data agrees well with the formula in all the dwell time plots reported in this article.

8) Save the figures and the result file, if completed successfully, or an error log file:
Finally, all of the intermediate steps are saved in figures (png format) and the results are saved in a result.txt file. If an error occurs during any of the steps, a log file error.txt describing the specific error message is automatically created for further investigation or debugging.

Info.txt:
* time_interval: time interval between frame in seconds
* spot_size: size of diffraction limited spot in pixels
* drift_correct: True or False for drift correction
* flatfield_correct: True for False for flatfield correction
* frame_offset: cutoff in frame number to exclude short dwell events
* save_trace_num: the number of trace figures to be saved as examples
* intensity_min_num: the number of groups in the minimum intensity distribution from the spot traces
* intensity_min_index: the index of group to be selected in the minimum intensity distribution from the spot traces
* intensity_min_cutoff: a multiplication factor to SD to include inliers in the minimum intensity distribution from the spot traces
* intensity_max_num: the number of groups in the maximum intensity distribution from the spot traces
* intensity_max_index: the index of group to be selected in the maximum intensity distribution from the spot traces
* intensity_max_cutoff: multiplication factor to SD to include inliers in the maximum intensity distribution from the spot traces
* HMM_RMSD_cutoff: multiplication factor to SD to include inliers in the RMSD of HMM fitting to the trace
* HMM_unbound_cutoff: multiplication factor to SD to include inliers in the unbound state intensity from the HMM fitting
* HMM_bound_num: the number of groups in the bound state intensity distribution from HMM fitting
* HMM_bound_index: the index of group to be selected in the bound state intensity distribution from HMM fitting
* HMM_bound_cutoff: multiplication factor to SD to include inliers in the bound state intensity from the HMM fitting

Code availability:
The analysis code is written in Python 3 and available in a Github repository (https://github.com/jmsung/smor-analysis). Follow the instructions in the repository for installation. The code is compatible with several operating systems including Windows, OSX, and Linux. Please contact Jongmin Sung (jongminsung@gmail.com) for suggestions or bug reports.
