# Supplementary material for: Single-molecule analysis of specificity and multivalency in binding of short linear substrate motifs to the APC/C
Source: Nat Commun. 2022 Jan 17;13:341. doi: 10.1038/s41467-022-28031-2 (PMC8764033; doi:10.1038/s41467-022-28031-2)
Supplement: Supplementary file 3 — Description of Additional Supplementary Files [file 41467_2022_28031_MOESM3_ESM.pdf]

Description of Additional Supplementary Files

File Name: Supplementary Movie 1
Description: Movie of Cy5-labeled Hsl1 D box peptide binding to Cdh1^{WD40} (see Fig. 2c). The movie was made using a 100 ms imaging interval at 10 fps (real time) for 100 frames.

File Name: Supplementary Movie 2
Description: Movie of Cy5-labeled Hsl1 D box mutant peptide binding to Cdh1^{WD40} (see Fig. 2d). The movie was made using a 100 ms imaging interval at 10 fps (real time) for 100 frames.

File Name: Supplementary Movie 3
Description: Movie of Cy5-labeled Hsl1 D box peptide binding to APC/C^{apo} (see Fig. 2e). The movie was made using a 32 ms imaging interval at 10 fps (real time) for 100 frames.

File Name: Supplementary Movie 4
Description: Movie of Cy5-labeled Hsl1 D box peptide binding to APC/C^{Cdh1} (see Fig. 2f). The movie was made using a 5 s imaging interval at 10 fps (real time) for 100 frames.

File Name: Supplementary Movie 5
Description: Movie of Cy5-labeled Hsl1 D box peptide binding to APC/C^{Cdc20} (see Fig. 2g). The movie was made using a 60 ms imaging interval at 10 fps (real time) for 100 frames.
