# Supplementary material for: Single-molecule analysis of specificity and multivalency in binding of short linear substrate motifs to the APC/C
Source: Nat Commun. 2022 Jan 17;13:341. doi: 10.1038/s41467-022-28031-2 (PMC8764033; doi:10.1038/s41467-022-28031-2)
Supplement: Supplementary file 9 — Reporting Summary [file 41467_2022_28031_MOESM9_ESM.pdf]

nature portfolio

Corresponding author(s): David O. Morgan

Last updated by author(s): Dec 21, 2021

Reporting Summary

Nature Portfolio wishes to improve the reproducibility of the work that we publish. This form provides structure for consistency and transparency in reporting. For further information on Nature Portfolio policies, see our Editorial Policies and the Editorial Policy Checklist.

Statistics

For all statistical analyses, confirm that the following items are present in the figure legend, table legend, main text, or Methods section.

n/a	Confirmed	
☐	☒	The exact sample size (*n*) for each experimental group/condition, given as a discrete number and unit of measurement
☐	☒	A statement on whether measurements were taken from distinct samples or whether the same sample was measured repeatedly
☒	☐	The statistical test(s) used AND whether they are one- or two-sided<br>*Only common tests should be described solely by name; describe more complex techniques in the Methods section.*
☒	☐	A description of all covariates tested
☒	☐	A description of any assumptions or corrections, such as tests of normality and adjustment for multiple comparisons
☐	☒	A full description of the statistical parameters including central tendency (e.g. means) or other basic estimates (e.g. regression coefficient) AND variation (e.g. standard deviation) or associated estimates of uncertainty (e.g. confidence intervals)
☒	☐	For null hypothesis testing, the test statistic (e.g. *F*, *t*, *r*) with confidence intervals, effect sizes, degrees of freedom and *P* value noted<br>*Give P values as exact values whenever suitable.*
☒	☐	For Bayesian analysis, information on the choice of priors and Markov chain Monte Carlo settings
☒	☐	For hierarchical and complex designs, identification of the appropriate level for tests and full reporting of outcomes
☒	☐	Estimates of effect sizes (e.g. Cohen's *d*, Pearson's *r*), indicating how they were calculated

Our web collection on statistics for biologists contains articles on many of the points above.

Software and code

Policy information about availability of computer code

Data collection	Collection of microscopy images was performed using microManager software version 2.0 (https://micro-manager.org).
Data analysis	Binding data were analyzed with GraphPad Prism version 8.4.<br>Image analysis was performed with custom code described in Supplementary Note 1 and deposited at a public repository (https://github.com/jmsung/smor-analysis).

For manuscripts utilizing custom algorithms or software that are central to the research but not yet described in published literature, software must be made available to editors and reviewers. We strongly encourage code deposition in a community repository (e.g. GitHub). See the Nature Portfolio guidelines for submitting code & software for further information.

Data

Policy information about availability of data

All manuscripts must include a data availability statement. This statement should provide the following information, where applicable:

- Accession codes, unique identifiers, or web links for publicly available datasets
- A description of any restrictions on data availability
- For clinical datasets or third party data, please ensure that the statement adheres to our policy

All video and single-molecule analysis data generated in this study are available on a public repository (https://zenodo.org/record/5726046). Source data for Fig.1 and Supplementary Figs. 1, 2, 5, 6 are provided with this paper.

Field-specific reporting

Please select the one below that is the best fit for your research. If you are not sure, read the appropriate sections before making your selection.

[x] Life sciences [ ] Behavioural & social sciences [ ] Ecological, evolutionary & environmental sciences

For a reference copy of the document with all sections, see nature.com/documents/nr-reporting-summary-flat.pdf

Life sciences study design

All studies must disclose on these points even when the disclosure is negative.

Sample size	As is the standard in the field, at least two independent videos were obtained for each ligand-protein combination. The list of replicate movies is provided in Supplementary Table 1. For each independent replicate movie, the standard in the field is to analyze at least 100 binding events, but we analyzed several hundred and sometimes thousands of events per movie, resulting in very small errors in mean dwell times.
Data exclusions	No data exclusions.
Replication	All experiments and video analyses were replicated independently at least once, demonstrating the reproducibility of all results.
Randomization	Our study involves biochemical analyses of purified proteins. It does not include statistical comparisons between groups of subjects or other samples. Thus, sample or subject randomization is not applicable to this work.
Blinding	Our study does not involve statistical comparisons of groups of experimental subjects or other samples, and therefore blinding is not applicable.

Reporting for specific materials, systems and methods

We require information from authors about some types of materials, experimental systems and methods used in many studies. Here, indicate whether each material, system or method listed is relevant to your study. If you are not sure if a list item applies to your research, read the appropriate section before selecting a response.

Materials & experimental systems

n/a	Involved in the study	
[ ]	[x]	Antibodies
[x]	[ ]	Eukaryotic cell lines
[x]	[ ]	Palaeontology and archaeology
[x]	[ ]	Animals and other organisms
[x]	[ ]	Human research participants
[x]	[ ]	Clinical data
[x]	[ ]	Dual use research of concern

Methods

n/a	Involved in the study	
[x]	[ ]	ChIP-seq
[x]	[ ]	Flow cytometry
[x]	[ ]	MRI-based neuroimaging

Antibodies

Antibodies used	Biotin-conjugated mouse monoclonal anti-Strep-Tag II antibody (LSBio, Cat. No. LS-C203632-100).<br>Biotin-conjugated rabbit polyclonal anti-GFP antibody (Rockland, Cat. No. 600-406-215).
Validation	Specificity of both antibodies was confirmed by control experiments showing minimal background with antibody alone in all experiments, presented in Supplementary Figs. 3, 7, 8.
